# Characterization and spoilage potential of *Bacillus cereus* isolated from farm environment and raw milk

**DOI:** 10.3389/fmicb.2022.940611

**Published:** 2022-09-14

**Authors:** Lu Meng, Ruirui Zhang, Lei Dong, Haiyan Hu, Huimin Liu, Nan Zheng, Jiaqi Wang, Jianbo Cheng

**Affiliations:** ^1^Laboratory of Quality and Safety Risk Assessment for Dairy Products of Ministry of Agriculture and Rural Affairs, Institute of Animal Sciences, Chinese Academy of Agricultural Sciences, Beijing, China; ^2^Key Laboratory of Quality & Safety Control for Milk and Dairy Products of Ministry of Agriculture and Rural Affairs, Institute of Animal Sciences, Chinese Academy of Agricultural Sciences, Beijing, China; ^3^College of Animal Science and Technology, Anhui Agricultural University, Hefei, China; ^4^Precision Livestock and Nutrition Laboratory, Teaching and Research Centre (TERRA), Gembloux Agro-Bio Tech, University of Liège, Gembloux, Belgium

**Keywords:** *B. cereus sl*, MLST, toxin genes, proteolytic activity, dairy farm

## Abstract

*Bacillus cereus sensu lato* (*B. cereus sl*) is important spoilage bacteria causing milk structure and flavor changes and is ubiquitous in the environment. This study addresses the biodiversity, toxicity, and proteolytic activity of *B. cereus sl* from 82 environmental samples and 18 raw bovine milk samples from a dairy farm in the region of Tianjin. In sum, 47 *B. cereus sl* isolates were characterized through biochemical tests, 16S rRNA gene sequencing, and *panC* gene analysis. Fourteen sequence types (STs) of *B. cereus sl* were found in raw bovine milk samples, and five new STs (ST2749, ST2750, ST2751, ST2752, and ST2753) were identified in this study. ST1150 was the dominant ST, associated with fecal, air, drinking water, teat skin, teat cup, and teat dip cup. The results of toxin gene analyses showed that 12.77% and 8.51% of isolates carried *hbl*ACD and *nhe*ABC operons, respectively. In addition, the detection rate of emetic *ces*B gene was 21.28%. *B. cereus sl* demonstrated high spoilage potentials even at 7°C, which has the proteolytic activity of 14.32 ± 1.96 μmol of glycine equivalents per ml. Proteolytic activities were significantly (*p* < 0.05) decreased after the heat treatment. The residual activity of protease produced at 7°C was significantly higher than that produced at 25°C and 37°C after treatment at 121°C for 10 s and 135°C for 5 s (*p* < 0.01). Together, the results provide insights into the characteristics of *B. cereus sl* from farm environment and raw bovine milk and revealed that *B. cereus sl* contamination should also be monitored in raw milk for ultra-high temperature (UHT) products. This knowledge illustrates that strict cleaning management should be implemented to control *B. cereus sl* and assure high-quality milk products.

## Introduction

*Bacillus cereus sensu lato* (*B. cereus sl*) is a bacterial group that can be commonly detected in environment samples such as soil (Mendez Acevedo et al., 2020), water (Brillard et al., 2015), and in various foods like retail aquatic products (Zhang et al., 2020), raw milk and pasteurized milk (Liu et al., 2020; Radmehr et al., 2020), vegetables (Yu et al., 2019a), and ready-to-eat foods (Yu et al., 2019b). *B. cereus sl* group consists of seven species, such as *B. anthracis, B. cytotoxicus, B. mycoides, B. pseudomycoides, B. cereus sensu stricto (ss), B. thuringiensis*, and *B. weihenstephanensis* (Kumari and Sarkar, 2016).

In European Union countries, more than 500 cases of foodborne diseases confirmed each year were caused by *B. cereus sl* (European Food Safety Authority (EFSA) and European Centre for Disease Prevention and Control (ECDPC), 2016; Messelhäußer and Ehling-Schulz, 2018). *B. cereus sl* could induce two types of food poisoning: emetic and diarrheal (Granum and Lund, 1997; Sanchez-Chica et al., 2021). The emetic type, which results in vomiting, is always caused by a heat-stable cereulide toxin that is produced by growing cells in food. The emetic strains are often linked to pasta and rice, which are contaminated with emetic *B. cereus ss* and high levels of cereulide (Shiota et al., 2010; Naranjo et al., 2011). The diarrheal type is always caused by enterotoxins, which are always related to milk/milk products (Kramer and Gilbert, 1989; Granum and Lund, 1997).

*Bacillus cereus sl* contaminants not only threaten human health but also cause dairy product spoilage because many strains are psychrotrophic (Porcellato et al., 2021). It is one of two dominant species in the dairy plant environment and milk products (Lucking et al., 2013). As a spore-forming bacterial group, *B. cereus sl* is resistant to heat and survives in pasteurized milk and ultra-high temperature (UHT) milk (Alonso et al., 2021). In addition, it can form biofilms on milk tanks and piping systems in dairy plants, driving higher resistance to heat and cleaning-in-place (CIP) procedures (Faille et al., 2014). Porcellato et al. (2018) observed that levels of *B. cereus* increased in pasteurized milk stored at 8°C to become the predominant bacteria in the milk. Apart from this, *B. cereus sl* affects the organoleptic quality of milk and milk products since it produces extracellular proteases (Kumari and Sarkar, 2016). The production of proteases could induce changes in structure and flavors, such as “sweet curdling” defects (Lucking et al., 2013).

*Bacillus cereus sl* has been identified as a considerable spoilage bacterium in the dairy industry, and the spores are found contaminating raw milk primarily during milking because they are ubiquitously in the dairy farm (Gopal et al., 2015). Therefore, this research aimed to investigate the spoilage and toxigenic potential of *B. cereus sl* from farm environment and raw bovine milk in Tianjin, China. The combination of proteolytic activity and toxigenic potential of different *B. cereus sl* multilocus sequence typing (MLST) isolates would help to evaluate the population dynamics in China's dairy farm.

## Materials and methods

### Sample collections

A total of 100 samples from a dairy farm in Tianjin, China were collected from September to November 2019. They included 82 environmental samples (nine fecal samples, three bedding samples, three air samples, nine drinking water samples, nine teat skin samples, nine teat cup samples, nine pre-dip solution samples, nine post-dip solution samples, nine pre-dip cup samples, nine post-dip cup samples, three feed samples, and one spray water sample) and 18 raw milk samples. All the samples were collected according to Du et al. (2020). Then, all the samples were stored at −20°C before DNA extraction.

### Isolation and characterization of *B. cereus sl* strains

For *B. cereus sl* isolation, all the samples were diluted and first chosen according to the National Food Safety Standard Method for Food Microbiological Examination (GB 4789.40-2010) (Gao et al., 2018). In total, 166 colonies have been picked. These 166 colonies were picked and then placed onto the Mannitol-Yolk Polymyxin Agar Plate (Beijing Land Bridge Technology Co. Ltd., Beijing, China), and then cultured at 30°C for 24 h (Owusu-Kwarteng et al., 2017). The colonies with pink color (81/166, 48.80%) were selected for further identification.

Identification of *B. cereus sl* isolates used a polymerase chain reaction (PCR) thermal cycler (Bio-Rad S1000; Bio-Rad, Hercules, CA, USA) *via* 16S rRNA and *panC* gene. DNA of *B. cereus* isolates was extracted with the InstaGene Matrix DNA Extraction Kit (Bio-Rad) following the instructions of Owusu-Kwarteng et al. (2017). The *panC* gene analysis was performed using the primers (Sangon, China), and conditions are shown in Supplementary Table S1 with negative and positive (DNA of *B. cereus* ATCC 11778; China Center of Industrial Culture Collection, Beijing, China) controls.

The PCR products were sequenced by SinoGenoMax Co. Ltd. Then, the sequencing results were BLASTed in the GenBank database (http://www.ncbi.nlm.nih.gov/BLAST/). The degree of homology for the universal 16S rRNA gene and the *panC* gene was higher than 99%.

### Multilocus sequence typing analysis

The MLST scheme was performed following a previous report (Yang et al., 2017). A total of seven housekeeping genes (*glpF, gmk, ilvD, pta, pur, pycA*, and *tpi*) were chosen according to the *B. cereus* MLST database (http://pubmlst.org/bcereus/) as described by Zahner et al. (2013). All the primers (Sangon, China) and PCR conditions are shown in Supplementary Table S1. PCR products were sequenced by SinoGenoMax Co. Ltd.

### Phylogenetic analysis

After the seven housekeeping gene sequences were spliced in order, the molecular evolutionary genetics analysis (MEGA, version 10.1.8) software was used for the phylogenetic relationship analysis (Fei et al., 2015). The ordered gene sequences were BLASTed first and then 13 standard strains (*B. anthracis* ATCC 14578, *B. cereus* ATCC 14579, *B*. cereus strain 09, *B. cereus* 30090, *B. cereus* strain Co1-1, *B. cereus* strain FORC60, *B. cereus* strain M3, *B. cereus* Q1, *B. mycoides* ATCC 6462, *B. paranthracis* strain BC307, *B. pseudomycoides* DSM 12442, *B. toyonensis* strain BV-17, and *B. weihenstephanensis* DSM 11821) were selected as specific reference strains in this study.

### Virulence genes

Eight virulence genes have been detected, including seven enterotoxigenic genes (*hbl*A, *hbl*C, *hbl*D, *nhe*A, *nhe*B, *nhe*C, and *cytK*-2) and an emetic gene (*ces*B). All the primers (Sangon, China) and PCR conditions are also shown in Supplementary Table S1. Electrophoresis using a 1.5% agarose gel was used to analyze the amplified fragments. Negative and positive controls were included.

### Phenotype of proteolytic activity

*Bacillus cereus sl* isolates were streaked on nutrient agar (Beijing Land Bridge Technology Co. Ltd.) supplemented with UHT milk [10%, vol/vol, Granarolo S.p.A., Bologna, Italy] (Meng et al., 2017). The plates were incubated at 7°C, 25°C, and 37°C for 7 days. The plates were monitored daily for transparent zones around the inoculated areas. Hydrolysis of milk was indicated by clear proteolytic halos (Porcellato et al., 2021).

### Proteolytic activity quantification

The *B. cereus sl* isolates that were positive for proteolytic halos were chosen for the quantification of proteolytic activity following the procedures of De Jonghe et al. (2010). The isolates were first cultured in nutrient broth (Beijing Land Bridge Technology Co. Ltd.) at 37°C for 18 h with 150 r min^−1^ shaking. Then, the cultures were diluted to a concentration of 1 × 10^5^ CFU ml^−1^ in 50-ml skim UHT milk (Granarolo S.p.A.) and cultured at 37°C to produce protease. After 7 days, all incubated samples were immediately centrifuged at 12,000 × *g* for 20 min at 4°C. About 1-ml supernatant was added to 9-ml skim UHT milk (Granarolo S.p.A.) followed by the addition of bronopol (Sinopharm Chemical Reagent Co., Ltd, Shanghai, China) and sodium azide (China Chemical Factory, Beijing, China) at final concentrations of 0.025% and 0.01%, respectively, to prevent the growth of bacteria. Finally, the samples were then incubated at 7°C, 25°C, and 37°C for another 14 days, respectively.

In order to quantify proteolytic activity, the trinitrobenzenesulfonic acid (TNBS) method was performed according to Meng et al. (2017) to track the existence of free α-amino groups. The protease activities of *B. cereus sl* isolates were calculated according to a standard curve and expressed as a concentration of glycine.

### Heat resistance of protease

About 2 ml of protease supernatant was added to a glass tube and heated at 55°C for 5 min, 95°C for 5 min, 121°C for 10 s, or 135°C for 5 s. All the samples were immediately cooled with ice. Then, 1 ml of heated protease supernatant was diluted with 9 ml of skim UHT milk (Granarolo S.p.A.) and incubated at 7°C, 25°C, and 37°C for 14 days, respectively.

### Statistical analyses

All data were analyzed *via* GraphPad Prism 9.0 (San Diego, CA, USA) through the one-way analysis of variance (ANOVA). The results were presented as the mean ± standard error of mean (SEM).

## Results

### Prevalence of *B. cereus sl* isolated from raw bovine milk and environmental samples from a dairy farm in Tianjin city

In total, 47 strains of *B. cereus sl* from 43 samples (43/100, 43.00%) were identified according to the biochemical experiments (Supplementary Table S2) and PCR results. An overview of the 47 isolates is shown in Table 1. Of these 47 isolates, the highest number of *B. cereus sl* was found in fecal samples (13 isolates), followed by raw milk samples (11 isolates), and post-dip cup samples (5 isolates).

**Table 1:** Prevalence of *B. cereus sl* isolated from raw milk and dairy farm environment.

**Sample sources**	**No. of samples**	**No. of *B. cereus sl***	**Isolates**	**STs**
Milk	18	11	13-M1	1150
			14-M2	1013
			15-M3	281
			16-M4	205
			24-M5	1150
			25-M6	127
			43-M7	1150
			44-M8	2751
			45-M9	1419
			46-M10	1150
			47-M11	1150
Fecal	9	13	6-F1	1943
			7-F2	1419
			8-F3	1150
			9-F4	1943
			10-F5	127
			11-F6	2749
			12-F7	2750
			22-F8	1419
			23-F9	127
			39-F10	1150
			40-F11	1150
			41-F12	1150
			42-F13	1150
Bedding	3	2	27-DL1	142
			28-DL2	1013
Air	3	2	17-A1	1150
			21-A2	1150
Drinking water	9	3	33-D1	744
			34-D2	2752
			35-D3	1150
Teat skin	9	3	36-RT1	1150
			37-RT2	1150
			38-RT3	744
Teat cup	9	2	5-NB1	1150
			32-NB2	205
Pre-dip solution	9	1	19-QYY1	1150
Post-dip solution	9	2	1-HYY1	1150
			18-HYY2	2753
Pre-dip cup	9	5	2-QYB1	1150
			3-QYB2	1150
			29-QYB3	281
			30-QYB4	1150
			31-QYB5	205
Post-dip cup	9	3	4-HYB1	1150
			20-HYB2	744
			26-HYB3	205

### The diversity of *B. cereus sl*

The novel sequences and STs were submitted onto the PubMLST database (Priest et al., 2004), which clustered the isolates into 14 *B. cereus sl* STs based on the allelic coding of the seven housekeeping genes and the MLST database reference (http://pubmlst.org/bcereus/) (Yang et al., 2017). Five novel STs were detected (Table 1).

The predominant ST was ST1150 (22/47, 46.80%), followed by ST205 (4/47, 8.51%), ST1419 (3/47, 6.38%), ST744 (3/47, 6.38%), ST127 (3/47, 6.38%), ST281 (2/47, 4.26%), ST1013 (2/47, 4.26%), ST1943 (2/47, 4.26%), ST2749 (1/47, 2.13%), ST2750 (1/47, 2.13%), ST2751 (1/47, 2.13%), ST2752 (1/47, 2.13%), ST2753 (1/47, 2.13%), and ST142 (1/47, 2.13%). Moreover, ST1150 was isolated from ten different types of samples, including milk, fecal, air, drinking water, teat skin, teat cup, pre-dip solution, post-dip solution, pre-dip cup, and post-dip cup. The new STs were numbered from 2749 to 2753, which were from fecal (ST2749 and ST2750), milk (ST2751), drinking water (ST2752), and post-dip solution (ST2753), as shown in Table 1.

### Phylogenetic analysis

**Figure 1:**
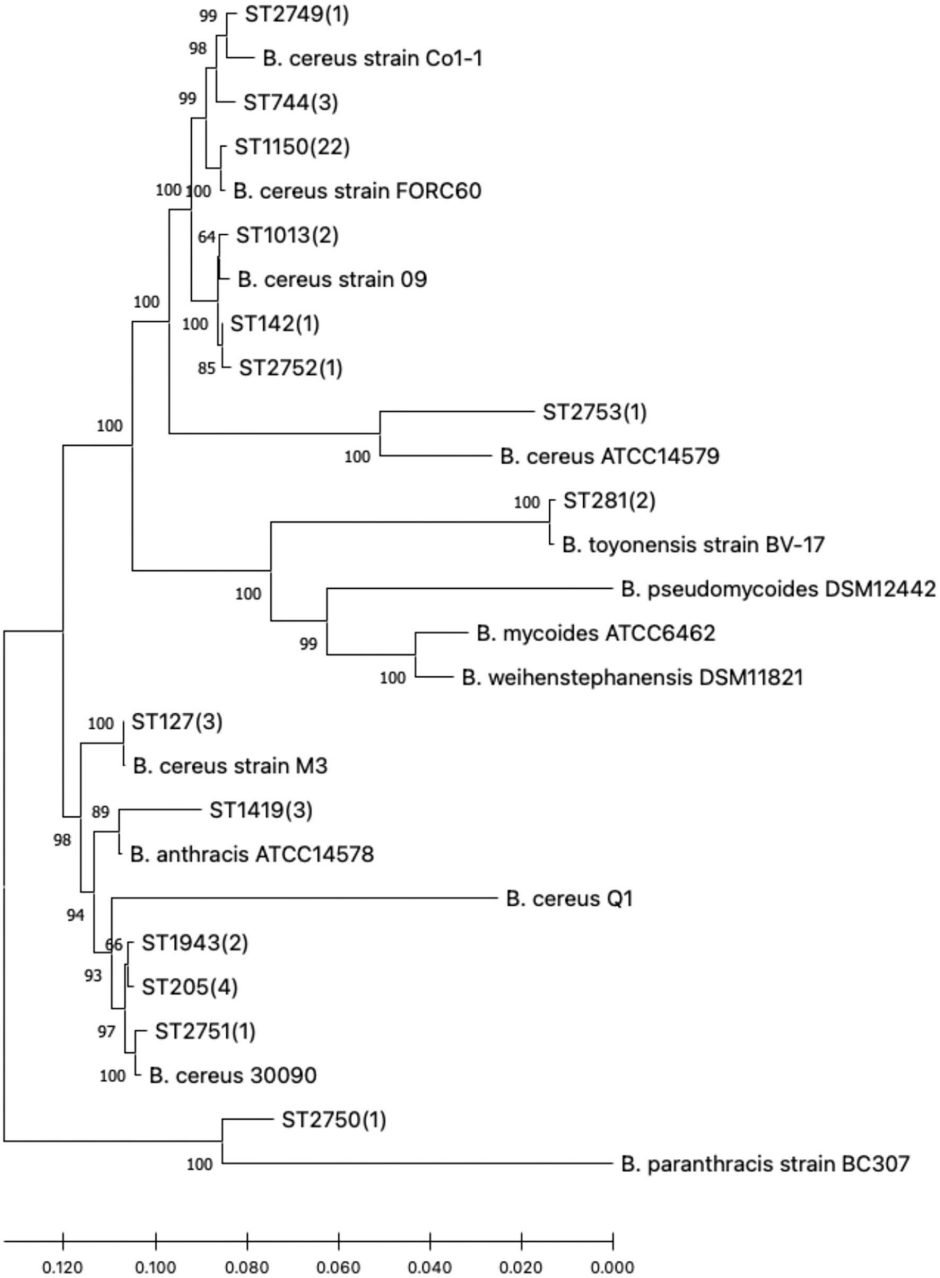
Phylogenetic tree of the 47 *B. cereus sl* isolates studied and reference strains. Numbers in parentheses indicate how many study isolates are associated with the sequence type (ST).

In view of the linkage sequences of the seven housekeeping genes of 47 *B. cereus sl*, a neighbor-joining phylogenetic tree was constructed. All the isolates in this research and the 13 selected reference strains showed a clear phylogenetic relationship as seen in Figure 1.

According to the MLST database, it was found that the gene sequences of these *B. cereus sl* STs with a close phylogenetic relationship differed only by a few bases. The novel ST, designated ST2749, ST2751, ST2752, and ST2753, had a closer phylogenetic relationship with *B. cereus* strains; however, ST2750 was close to *B. paranthracis*.

### Distribution of virulence genes

The virulence genes are divided into hemolytic enterotoxin complex genes (*hbl*ACD operon), nonhemolytic enterotoxin complex genes (*nhe*ABC operon), cytotoxin K (*cyt*K-2), and cereulide (*ces*B) (Owusu-Kwarteng et al., 2017). The virulence gene distribution among 47 *B. cereus sl* isolates is listed in Table 2 and Supplementary Table S3. The detection rates of *nhe*B and *nhe*C were the highest; both were 97.87%, followed by *hbl*A (59.57%) and *hbl*C (57.45%). However, only 12.77% (*n* = 6) and 8.51% (*n* = 4) isolates carried *hbl*ACD and *nhe*ABC gene clusters, respectively. No virulence genes were detected in one isolate (2.13%). Furthermore, ten strains (21.28%) of *B. cereus sl* carried the virulence gene *ces*B together with hemolytic and/or nonhemolytic enterotoxin complex genes.

**Table 2:** Distribution of virulence genes in *B. cereus sl*.

**Patterns**	***hbl*A**	***hbl*C**	***hbl*D**	***nhe*A**	***nhe*B**	***nhe*C**	***cyt*k**	***ces*B**	**No. of strains (%) for target pattern (Total = 47)**
I	+	-	-	-	+	+	+	-	1 (2.13)
II	+	+	-	-	+	+	-	+	4 (8.51)
III	+	+	-	-	+	+	-	-	13 (27.66)
IV	+	+	-	-	+	+	+	-	1 (2.13)
V	+	+	-	+	+	+	-	-	1 (2.13)
VI	+	+	-	+	+	+	+	-	1 (2.13)
VII	+	+	+	-	+	+	-	-	6 (12.77)
VIII	-	-	-	+	+	+	+	-	1 (2.13)
IX	-	-	-	+	+	+	+	+	1 (2.13)
X	-	-	-	-	+	+	-	-	11 (23.4)
XI	-	-	-	-	+	+	-	+	4 (8.51)
XII	-	-	-	-	+	+	+	-	1 (2.13)
XIII	-	-	-	-	+	+	+	+	1 (2.13)
XIV	-	-	-	-	-	-	-	-	1 (2.13)
Numbers (%) (Total = 47)	27 (57.45)	26 (55.32)	6 (12.77)	4 (8.51)	46 (97.87)	46 (97.87)	7 (14.89)	10 (21.28)	47 (100)

In total, 14 different virulence patterns were detected in this study. Thirteen *B. cereus sl* isolates (27.66%) carried *hbl*A, *hbl*C, *nhe*B, and *nhe*C, and ten isolates (21.28%) carried only *nhe*B and *nhe*C. Isolates carrying only *hbl*A/C/D and *nhe*A/B/C were detected in 31 isolates (65.96%), which suggests that diarrheal strains were more common among isolates from raw bovine milk and dairy farm environmental samples.

### Proteolytic activity of identified *B. cereus sl*

The proteolytic activity of the 47 isolates was evaluated at 7°C, 25°C, and 37°C. The appearance of a clear zone or halo in milk agar was used to determine the phenotypic results. Halos were detected in 42 isolates (89.36%) at 25°C and 37°C. No halos were found on the milk agar at 7°C (Supplementary Table S4). Moreover, in five isolates without halos (ST142, *n* = 1; ST205, *n* = 1; ST1150, *n* = 2; and ST1419, *n* = 1), no *ces*B gene was detected.

**Figure 2:**
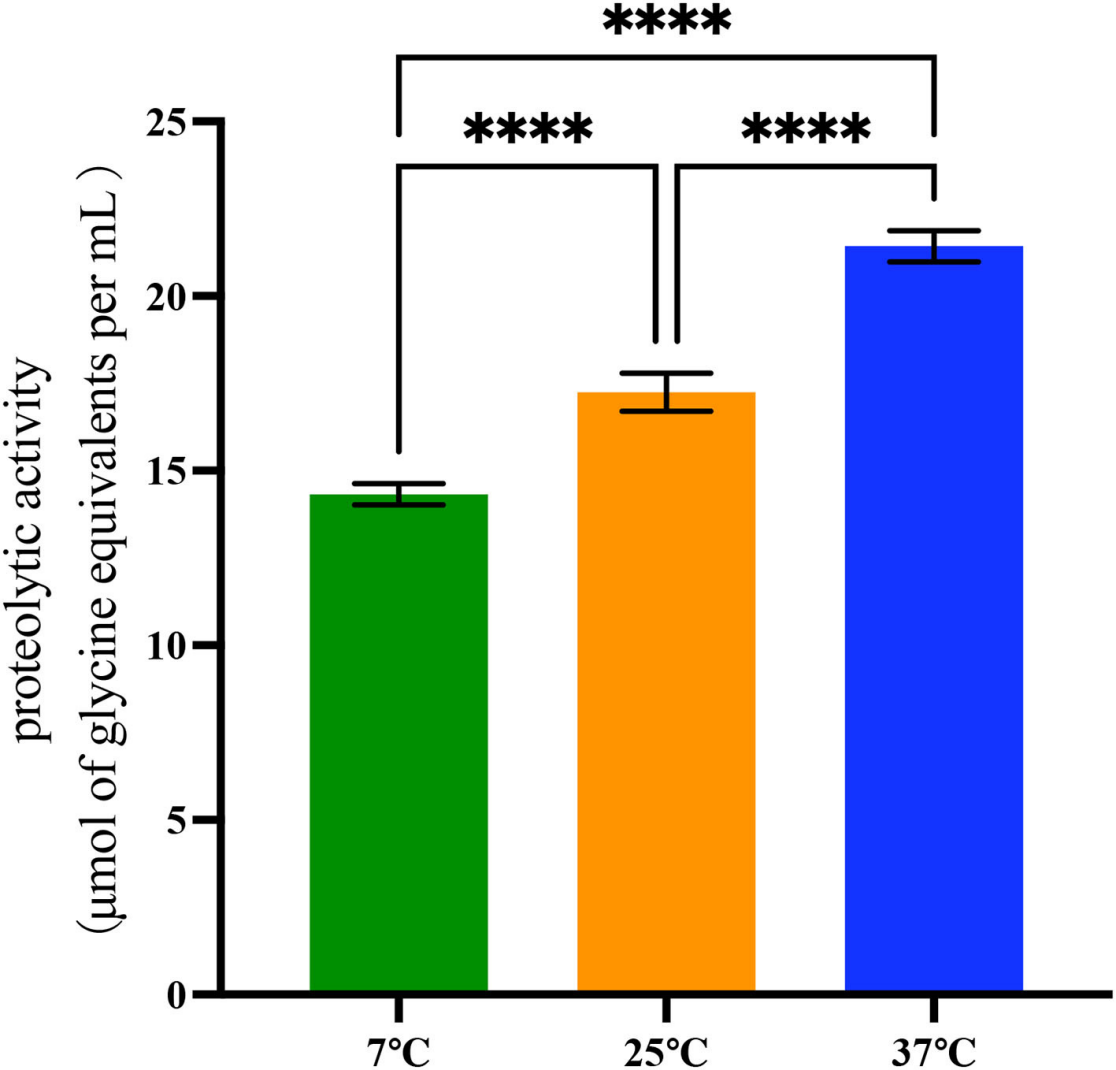
Proteolytic activity of 42 *B. cereus sl* strains at 7°C, 25°C, and 37°C. Data represent the mean of proteolytic activity ± SEM (****p < 0.0001).

The quantified proteolytic activity was performed in skim UHT milk (Figure 2 and Supplementary Table S4). First, a standard curve was built with glycine as the standard amino acid, and the *R*^2^ value was 0.998 (data not shown). Isolates were identified as proteolytically active when the value was above 2 μmol of glycine equivalents per ml.

Differences in proteolytic activity were found in proteases produced by 42 *B. cereus sl* isolates. At 37°C, the proteolytic activity of these isolates (21.43 ± 2.89 μmol of glycine equivalents per ml) was significantly higher than at 25°C and 7°C (*p* < 0.001). At 25°C, the proteolytic activity was 17.25 ± 3.56 μmol of glycine equivalents per ml, and it was 14.32 ± 1.96 μmol of glycine equivalents per ml at 7°C.

### Heat resistance of protease

**Figure 3:**
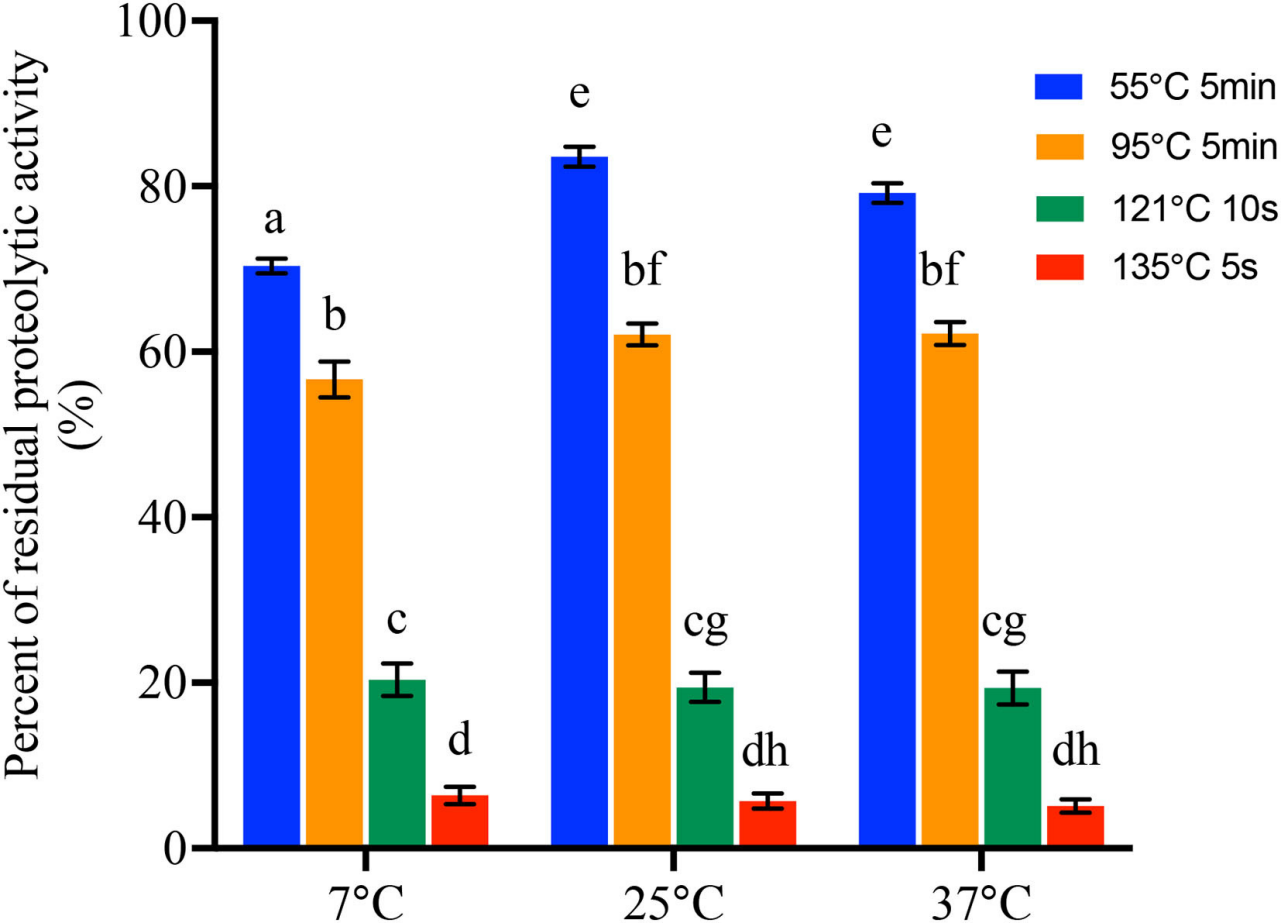
Percentage of residual proteolytic activity of the protease produced by 42 *B. cereus sl* isolates at 7°C, 25°C, and 37°C after treat at different temperatures and time. Data represent the mean of residual proteolytic activity percentage ± SEM. Significance compared with each other was represented as a–h (*p* < 0.01).

All the proteases produced by 42 *B. cereus sl* isolates at 7°C, 25°C, and 37°C were then heated to investigate the heat resistance at 55°C for 5 min, 95°C for 5 min, 121°C for 10 s, and 135°C for 5 s, respectively. In sum, all the proteases were resistant to 55°C for 5 min and 95°C for 5 min. However, after heating at 121°C for 10 s, proteolytic activity of 9, 9, and 10 isolates produced at 7°C, 25°C, and 37°C, respectively, was below 2 μmol of glycine equivalents per ml. Moreover, after heating at 135°C for 10 s, protease of 22, 21, and 21 isolates produced at 7°C, 25°C, and 37°C, respectively, was considered as no proteolytic activity (Supplementary Table S5). The percentages of residual proteolytic activity are shown in Figure 3 and Supplementary Table S5. There were significant differences in the residual proteolytic activity of *B. cereus sl* isolates between the treatment of 55°C for 5 min, 95°C for 5 min, 121°C for 10 s, and 135°C for 5 s (*p* < 0.001). The percentage of residual protease activity produced at 7°C was 13.40 ± 0.98%, 20.35 ± 12.74%, 56.65 ± 14.07%, and 70.36 ± 5.81% after 55°C for 5 min, 95°C for 5 min, 121°C for 10 s, and 135°C for 5 s, respectively. These values were 5.70 ± 5.93%, 19.45 ± 11.52%, 62.08 ± 8.64%, and 83.56 ± 7.80% for protease produced at 25°C and 5.09 ± 5.20%, 19.35 ± 12.80%, 62.19 ± 9.02%, and 79.18 ± 7.61% for protease produced at 37°C. Moreover, when the protease was heated at 55°C for 5 min, the percentage of residual proteolytic activity produced in the 25°C and 37°C groups was significantly higher than that for 7°C (*p* < 0.001).

## Discussion

In China, *B. cereus* foodborne outbreaks usually occurred in dairy-based products (Liu et al., 2020). Sporeformers, especially *B. cereus sl*, are considered important contaminants in the dairy environment and are related to milk quality and safety (Kumari and Sarkar, 2016; Huang et al., 2020). The *B. cereus* spore content of milk has been found to increase significantly during wet weather and is strongly associated with the contamination of the teats with soil (Christiansson et al., 1999). Characteristic of *B. cereus sl* makes it easier to contaminate raw milk and milk products from the environment. This leads to the reduction of shelf-life for milk products (Lucking et al., 2013; Rossi et al., 2018). Members of *B. cereus sl* can produce proteases, which degrade milk components and additives (Chen et al., 2003; Kumari and Sarkar, 2016). Therefore, this study has characterized *B. cereus sl* strains from raw milk and dairy environments and compared the proteolytic changes of different proteases after heat treatment.

Although morphological characteristics are used to visually distinguish colonies in a series of physiological and biochemical tests using conventional typing methods, it is still easy to introduce errors into the determination of the test results (Guinebretiere et al., 2008). The *panC* gene-encoded pantothenic acid-β-alanine ligase enzyme is related to the *B. cereus* cultural temperature and heat resistance (Kindle et al., 2019). Candelon et al. (2004) used *gdpD, panC*, and *plcR* gene analyses to distinguish and identify 12 strains of microorganisms from different *B. cereus*, including *B. cereus ss, B. anthracis, B thuringiensis*, and *B. weihenstephanensis* and showed that the *panC* gene had good typing ability. Guinebretiere et al. (2008) first analyzed the results of *panC* gene analysis using physiological and biochemical characteristics of strains. This test combines traditional methods with molecular typing methods (16S rRNA sequencing, specific gene *panC*, and MLST methods) to identify *B. cereus* from other *Bacillus* species. In this way, *B cereus* was distinguished from strains that were highly similar in both phenotype and 16S rRNA sequence (Vilas-Boas et al., 2007).

In this research, 47 *B. cereus sl* isolates were identified from 43 samples, with the highest occurrence in fecal samples (100.00%), followed by raw milk samples (61.11%). BioNumerics software analysis has illustrated that *B. cereus sl* isolates in raw milk might be from fecal, teat skin, teat cups, and dip cups (data not shown). The incident was similar to *B. cereus* prevalence on an Australian dairy farm, where *B. cereus* group was positive for 41% of 120 samples. Moreover, a high occurrence of *B. cereus* group was found in soil (93.0%, 13/14), feces (63.0%, 10/16), and raw milk (33.0%, 3/9) (McAuley et al., 2014). Around 72% (18/25) of soil from cattle-grazing farms and 46.6% (14/30) of raw milk samples were positive for *B. cereus sl* in Ghana (Owusu-Kwarteng et al., 2017). However, Cui et al. (2016) have identified a lower prevalence of *B. cereus* isolates from 10 local dairy farms in Beijing, China. Of the 306 milk and environmental samples, 88 *B. cereus* and four *B. thuringiensis* strains were isolated. *B. cereus* isolates were most common in liquid manure samples (100.0%, 8/8), followed by bedding (93.3%, 42/45), feces (78.9%, 15/19), feed (41.2%, 7/17), and raw milk (9.8%, 20/205). Fei et al. (2019) isolated only 2 (4%) *B. cereu*s strains from 50 raw bovine milk samples and 54 isolates (18%) from 300 environmental samples. In that study, bedding (8 isolates, 40%) and excrement (8 isolates, 40%) had the highest prevalence. *B. cereus* was most widely distributed at the *Bacillus* genus level from Korean dairy farms (Ryu et al., 2021), and a high prevalence of *B. cereus* was also found in raw milk (85%, 85/100) in Egypt (Hammad et al., 2021). Moreover, *B. cereus* strains also have been isolated and identified from buffalo raw milk in southwestern China, with a prevalence of 33.33% (50/150). The occurrence of *B. cereus sl* in this study has a similarity to other studies, which support the speculation that *Bacillus* spp. in raw milk is contaminated from the upper layer of soil in pastureland or from feces (Scheldeman et al., 2005; Ryu et al., 2021).

In this study, 47 strains of *B. cereus sl* were classified into 14 STs. Similarly, Fei et al. (2019) divided 54 *B. cereus* isolates into 18 STs, Zhuang et al. (2019) clustered 84 isolates into 24 STs, Zhao et al. (2020) divided 54 isolates into 24 STs, and Chang et al. (2021) classified 96 *B. cereus* isolates into 41 STs, illustrating that *B. cereus sl* from raw milk and farm environment has a high genetic diversity. We found ST1150 was the main ST of *B. cereus sl* among samples. However, in other studies, ST857 was the dominant ST in samples from air, bedding, cowsheds, feces, raw milk, pasteurized milk, UHT milk, silo tank, and ingredient tank samples (Lin et al., 2017; Fei et al., 2019; Zhao et al., 2020; Chang et al., 2021). ST26 has been found as another dominant *B. cereus* clonal complex. It was isolated from powder infant formula and cheese samples in China (Yang et al., 2017; Zhao et al., 2020). Neither ST857 nor ST26 were detected in our study. In addition, *B. cereus* ST142 and ST205, which have been detected in fecal, raw milk, teat cup, and drug bath cup samples, have also been found in cheese, pasteurized milk, cowsheds, fodder, and sole samples (Fei et al., 2019; Zhao et al., 2020). The differences in predominant subtypes among the studies demonstrated the diversity of STs at different locations (Lin et al., 2017).

The virulence genes *cyt*K, *hbl*ACD operon, and *nhe*ABC operon produce *B. cereus* enterotoxins, which have been reported as the main reason for diarrhea caused by food poisoning (Ngamwongsatit et al., 2008; Owusu-Kwarteng et al., 2017). Around 12.8% of strains harbored all three gene encoding for hemolytic enterotoxin HBL complex genes [*hbl*A, *hbl*C, and *hbl*D (*hbl*ACD)], which is similar to the presence (12.5%) in milk and milk products in Ghana (Owusu-Kwarteng et al., 2017). However, the prevalence rates of gene *hbl*ACD varied in degree. All three *hbl* genes (*hbl*A, *hbl*C and *hbl*D) were present in 51% *B. cereus sl* isolates from Norwegian bovine milk products (Porcellato et al., 2021). Around 45% of strains from pasteurized milk in China harbored enterotoxigenic *hbl*ACD genes (Gao et al., 2018). Moreover, Reis et al. (2013) found that 35.6% (23/63) of isolates from raw milk and milk products marketed in Brazil carried simultaneously *hbl*ACD genes. No strains harbored the *hbl*ABD gene cluster from two local dairy plants in China (Lin et al., 2017). Only 11.1% of the *B. cereus* strains isolated from 500 milk products in China carried the *hbl*ACD operon (Zhao et al., 2020). In addition, the *nhe*ABC operon had high prevalence rates in *B. cereus* strains. The detection rates of virulence gens *nhe*ABC were 100% (92/92) from 306 milk and environmental samples, 94.4% (51/54) from 500 dairy products (Zhao et al., 2020), 89.6% (86/96) from raw and pasteurized buffalo milk (Chang et al., 2021), and 74.07% (20/27) from two local dairy plants in China (Lin et al., 2017). However, in our study, evidence of the *nhe*ABC operon, by detection of *nhe*A, was detected in only four isolates. Mendelson et al. (2004) have revealed that NheA is not absolutely required for virulence in *B. anthracis*. The proportions of *cyt*K-2 in *B. cereus* in our study was 14.89%, which is lower than previously reported detection rates (Gao et al., 2018; Zhao et al., 2020). The *ces*B associated with emetic toxin was present in 21.2% of *B. cereus* strain in this study, which is higher than that isolated from milk and dairy farm environment in China and in Ghana, respectively (Cui et al., 2016; Owusu-Kwarteng et al., 2017) but lower than that from UHT milk processing lines (48.2%, Lin et al., 2017). Studies have found that the *hbl* and *ces* genes were related to the phylogenetic group (Okutani et al., 2019; Dietrich et al., 2021); however, no relationship was demonstrated in this study.

*Bacillus cereus sl* is considered to be the biological factor that causes food contamination and spoilage during the entire process of food and supplement production (Ehling-Schulz et al., 2019). Here, the proteolytic activity of the 42 isolates at 7°C, 25°C, and 37°C was significantly different, which is similar to that found by Yang et al. (2021). They found that protease activities were 14.40 and 29.70 U ml^−1^ at 10°C and 28°C, respectively. This indicated that *B. cereus* could produce protease at refrigeration temperature, and the enzyme activity increased significantly with the increase in temperature. Besides the heat-resistant spores, the protease produced by *B. cereus sl* also produces heat-resistant protease (Benahmed et al., 2020). In addition, *Bacillus* spp. produce a wide range of proteases, which have a higher proteolytic diversity compared to *Pseudomonas* spp., illustrating that *Bacillus* spp. would compromise milk quality and shorten shelf-life (Chen et al., 2003; Contesini et al., 2018; Porcellato et al., 2021).

## Conclusion

In this study, 47 *B. cereus sl* isolates were from dairy environmental and bovine milk samples, highlighting the diversity of *B. cereus sl* population present at the dairy farm. The MLST analysis demonstrated the high genetic diversity, with *B. cereus* ST1150 being the most common and widespread in the environment. Compared with *B. cereus* enterotoxins gene *cyt*K and enterotoxigenic genes (*hbl*ACD operon and *nhe*ABC operon), emetic gene *ces*B was only detected in 21.28% of the strains. Furthermore, proteases of *B. cereus sl* produced at low temperatures had higher resistance to UHT treatment. Therefore, from this study, an understanding of the hygienic conditions and control of *B. cereus sl* is needed to maintain milk quality and safety.

## Data availability statement

The original contributions presented in the study are included in the article/Supplementary material, further inquiries can be directed to the corresponding author.

## Author contributions

LM designed experiments and wrote the manuscript. RZ carried out experiments. LD and HH helped in the experiments. HL, JC, and NZ gave advice. JW gave opinions on the research design and supported the findings. All authors contributed to the article and approved the submitted version.

## Funding

This work was supported by the Project of Risk Assessment on Raw Milk (GJFP20220304), the Agricultural Science and Technology Innovation Program (ASTIP-IAS12), and the China Agriculture Research System of MOF and MARA (CARS36).

## Conflict of interest

The authors declare that the research was conducted in the absence of any commercial or financial relationships that could be construed as a potential conflict of interest.

## Publisher's note

All claims expressed in this article are solely those of the authors and do not necessarily represent those of their affiliated organizations, or those of the publisher, the editors and the reviewers. Any product that may be evaluated in this article, or claim that may be made by its manufacturer, is not guaranteed or endorsed by the publisher.
