# Supplementary material for: Characterization and spoilage potential of Bacillus cereus isolated from farm environment and raw milk
Source: Front Microbiol. 2022 Sep 14;13:940611. doi: 10.3389/fmicb.2022.940611 (PMC9514233; doi:10.3389/fmicb.2022.940611)
Supplement: Supplementary file 1 [file Table_1.DOCX]

**Supplemental Table 1. Primers information used in the work**

| **Category** | **Primer** | **Primer sequence** | **Annealing temp (ºC)** | **Amplicon size (bp)** | **Reference** |
| --- | --- | --- | --- | --- | --- |
| ***panC*** | ***panC*-F** | TYGGTTTTGTYCCAACRATGG | 55 | 651 | (Gdoura-Ben Amor et al., 2019) |
|  | ***panC*-R** | CATAATCTACAGTGCCTTTCG |  |  |  |
| **MLST** | ***glpF*-F** | GCGTTTGTGCTGGTGTAAGT | 59 | 372 | (Yang et al., 2017) |
|  | ***glpF*-R** | CTGCAATCGGAAGGAAGAAG |  |  |  |
|  | ***gmk*-F** | ATTTAAGTGAGGAAGGGTAGG | 56 | 504 |  |
|  | ***gmk*-R** | GCAATGTTCACCAACCACAA |  |  |  |
|  | ***ilvD*-F** | CGGGGCAAACATTAAGAGAA | 58 | 393 |  |
|  | ***ilvD*-R** | GGTTCTGGTCGTTTCCATTC |  |  |  |
|  | ***ilvD2**b-F^a^** | AGATCGTATTACTGCTACGG | 58 | 393 |  |
|  | ***ilvD2**b-R** | GTTACCATTTGTGCATAACGC |  |  |  |
|  | ***pta*-F** | GCAGAGCGTTTAGCAAAAGAA | 56 | 414 |  |
|  | ***pta*-R** | TGCAATGCGAGTTGCTTCTA |  |  |  |
|  | ***pur*-F** | CTGCTGCGAAAAATCACAAA | 56 | 348 |  |
|  | ***pur*-R** | CTCACGATTCGCTGCAATAA |  |  |  |
|  | ***pycA*-F** | GCGTTAGGTGGAAACGAAAG | 57 | 363 |  |
|  | ***pycA*-R** | CGCGTCCAAGTTTATGGAAT |  |  |  |
|  | ***tpi*-F** | GCCCAGTAGCACTTAGCGAC | 58 | 435 |  |
|  | ***tpi*-R** | CCGAAACCGTCAAGAATGAT |  |  |  |
| **Enterotoxigenic Genes** | ***Hbl*A-F** | GTGCAGATGTTGATGCCGAT | 55 | 237 | (Gao et al., 2018) |
|  | ***Hbl*A-R** | ATGCCACTGCGTGGACATAT |  |  |  |
|  | ***Hbl*C-F** | AATGGTCATCGGAACTCTAT | 55 | 386 |  |
|  | ***Hbl*C-R** | CTCGCTGTTCTGCTGTTAAT |  |  |  |
|  | ***Hbl*D-F** | AATCAAGAGCTGTCACGAAT | 55 | 436 |  |
|  | ***Hbl*D-R** | CACCAATTGACCATGCTAAT |  |  |  |
|  | ***Nhe*A-F** | TACGCTAAGGAGGGGCA | 55 | 475 |  |
|  | ***Nhe*A-R** | GTTTTTATTGCTTCATCGGCT |  |  |  |
|  | ***Nhe*B-F** | CTATCAGCACTTATGGCAG | 55 | 328 |  |
|  | ***Nhe*B-R** | ACTCCTAGCGGTGTTCC |  |  |  |
|  | ***Nhe*C-F** | CGGTAGTGATTGCTGGG | 55 | 557 |  |
|  | ***Nhe*C-R** | CAGCATTCGTACTTGCCAA |  |  |  |
|  | ***cyt*K-2-F** | AAAATGTTTAGCATTATCCGCTGT | 55 | 565 |  |
|  | ***cyt*K-2-R** | ACCAGTTGTATTAATAACGGCAATC |  |  |  |
| **Emetic Genes** | ***ces*B-F** | GGTGACACATTATCATATAAGGTG | 53 | 699 |  |
|  | ***ces*B-R** | GTAAGCGAACCTGTCTGTAACAACA |  |  |  |

^a^ *ilvD2*^*^ was an alternative to *ilvD* because *B. cereus* is an emetic toxin-producing strain. In this study, if the amplification of *ilvD* failed, then *ilvD2*^*^ was used.

Gao, T., Ding, Y., Wu, Q., Wang, J., Zhang, J., Yu, S., Yu, P., Liu, C., Kong, L., Feng, Z., Chen, M., Wu, S., Zeng, H., and Wu, H. (2018). Prevalence, virulence genes, antimicrobial susceptibility, and genetic diversity of *Bacillus cereus* isolated from pasteurized milk in China. *Front. Microbiol.* 9**,** 533.

Gdoura-Ben Amor, M., Jan, S., Baron, F., Grosset, N., Culot, A., Gdoura, R., Gautier, M., and Techer, C. (2019). Toxigenic potential and antimicrobial susceptibility of Bacillus cereus group bacteria isolated from Tunisian foodstuffs. *BMC Microbiol* 19**,** 196.

Yang, Y., Yu, X., Zhan, L., Chen, J., Zhang, Y., Zhang, J., Chen, H., Zhang, Z., Zhang, Y., Lu, Y., and Mei, L. (2017). Multilocus sequence type profiles of Bacillus cereus isolates from infant formula in China. *Food Microbiol* 62**,** 46-50.
