# Supplementary material for: Characterization and spoilage potential of Bacillus cereus isolated from farm environment and raw milk
Source: Front Microbiol. 2022 Sep 14;13:940611. doi: 10.3389/fmicb.2022.940611 (PMC9514233; doi:10.3389/fmicb.2022.940611)
Supplement: Supplementary file 2 [file Table_2.DOCX]

**Supplemental Table S2. Results of biochemical experiments**

| Isolated strain | **Experimental Project** | | | | | | | |
| --- | --- | --- | --- | --- | --- | --- | --- | --- |
|  | Motility | Lysozyme | Glucose | Voges-Proskauer | Nitrate | Mannitol | Gelatin | Simon's Citrate |
| 13-M1 | + | + | + | - | + | - | +/- | +/- |
| 14-M2 | + | + | + | - | + | - | +/- | +/- |
| 15-M3 | + | + | + | - | + | - | +/- | +/- |
| 16-M4 | + | + | + | - | + | - | +/- | +/- |
| 24-M5 | + | + | + | - | + | - | +/- | +/- |
| 25-M6 | + | + | + | - | + | - | +/- | +/- |
| 43-M7 | + | + | + | - | + | - | +/- | +/- |
| 44-M8 | + | + | + | - | + | - | +/- | +/- |
| 45-M9 | + | + | + | - | + | - | +/- | +/- |
| 46-M10 | + | + | + | - | + | - | +/- | +/- |
| 47-M11 | + | + | + | - | + | - | +/- | +/- |
| 6-F1 | + | + | + | - | + | - | +/- | +/- |
| 7-F2 | + | + | + | - | + | - | +/- | +/- |
| 8-F3 | + | + | + | - | + | - | +/- | +/- |
| 9-F4 | + | + | + | - | + | - | +/- | +/- |
| 10-F5 | + | + | + | - | + | - | +/- | +/- |
| 11-F6 | + | + | + | - | + | - | +/- | +/- |
| 12-F7 | + | + | + | - | + | - | +/- | +/- |
| 22-F8 | + | + | + | - | + | - | +/- | +/- |
| 23-F9 | + | + | + | - | + | - | +/- | +/- |
| 39-F10 | + | + | + | - | + | - | +/- | +/- |
| 40-F11 | + | + | + | - | + | - | +/- | +/- |
| 41-F12 | + | + | + | - | + | - | +/- | +/- |
| 42-F13 | + | + | + | - | + | - | +/- | +/- |
| 27-DL1 | + | + | + | - | + | - | +/- | +/- |
| 28-DL2 | + | + | + | - | + | - | +/- | +/- |
| 17-A1 | + | + | + | - | + | - | +/- | +/- |
| 21-A2 | + | + | + | - | + | - | +/- | +/- |
| 33-D1 | + | + | + | - | + | - | +/- | +/- |
| 34-D2 | + | + | + | - | + | - | +/- | +/- |
| 35-D3 | + | + | + | - | + | - | +/- | +/- |
| 36-RT1 | + | + | + | - | + | - | +/- | +/- |
| 37-RT2 | + | + | + | - | + | - | +/- | +/- |
| 38-RT3 | + | + | + | - | + | - | +/- | +/- |
| 5-NB1 | + | + | + | - | + | - | +/- | +/- |
| 32-NB2 | + | + | + | - | + | - | +/- | +/- |
| 19-QYY1 | + | + | + | - | + | - | +/- | +/- |
| 1-HYY1 | + | + | + | - | + | - | +/- | +/- |
| 18-HYY2 | + | + | + | - | + | - | +/- | +/- |
| 2-QYB1 | + | + | + | - | + | - | +/- | +/- |
| 3-QYB2 | + | + | + | - | + | - | +/- | +/- |
| 29-QYB3 | + | + | + | - | + | - | +/- | +/- |
| 30-QYB4 | + | + | + | - | + | - | +/- | +/- |
| 31-QYB5 | + | + | + | - | + | - | +/- | +/- |
| 4-HYB1 | + | + | + | - | + | - | +/- | +/- |
| 20-HYB2 | + | + | + | - | + | - | +/- | +/- |
| 26-HYB3 | + | + | + | - | + | - | +/- | +/- |
